# Exome sequencing of case-unaffected-parents trios reveals recessive and *de novo* genetic variants in sporadic ALS

**DOI:** 10.1038/srep09124

**Published:** 2015-03-16

**Authors:** Karyn Meltz Steinberg, Bing Yu, Daniel C. Koboldt, Elaine R. Mardis, Roger Pamphlett

**Affiliations:** 1 The Genome Institute, Washington University School of Medicine, St. Louis, MO, USA; 2 Department of Medical Genomics, Royal Prince Alfred Hospital and Sydney Medical School, The University of Sydney, Sydney, New South Wales, Australia; 3 The Stacey MND Laboratory, Department of Pathology, The University of Sydney, Sydney, New South Wales, Australia

## Abstract

The contribution of genetic variants to sporadic amyotrophic lateral sclerosis (ALS) remains largely unknown. Either recessive or *de novo* variants could result in an apparently sporadic occurrence of ALS. In an attempt to find such variants we sequenced the exomes of 44 ALS-unaffected-parents trios. Rare and potentially damaging compound heterozygous variants were found in 27% of ALS patients, homozygous recessive variants in 14% and coding *de novo* variants in 27%. In 20% of patients more than one of the above variants was present. Genes with recessive variants were enriched in nucleotide binding capacity, ATPase activity, and the dynein heavy chain. Genes with *de novo* variants were enriched in transcription regulation and cell cycle processes. This trio study indicates that rare private recessive variants could be a mechanism underlying some case of sporadic ALS, and that *de novo* mutations are also likely to play a part in the disease.

An intensive search for genetic disorders that could underlie amyotrophic lateral sclerosis (ALS) has uncovered pathogenetic variants in about 10% of sporadic ALS (SALS) and 60% of familial ALS (FALS) patients^1^. While this represents remarkable progress in only a few years, a major question is whether most SALS arises from environmental factors, genetic predisposition, or some combination of the two. Attempts have been made to look for environmental factors or gene-environment interactions underlying ALS in, for example, pesticide exposure^2^, but despite work from many research groups no convincing environmental factor for ALS has been found. Furthermore, numerous genome-wide association studies (GWAS) have revealed no reproducible findings of common variants that would lead to ALS susceptibility in a substantial proportion of patients^3^. There could be a number of reasons for such negative results in GWAS, one being a mismatch of exposure to environmental factors and the presence of susceptibility genes. While there may be an environmental contribution to SALS, the genetic contribution could come mostly from rare variants, which still allows for strong gene-environment interactions.

If SALS has a strong genetic component, consideration needs to be given to the genetic mechanisms that could be responsible for the sporadic occurrence of most cases of ALS. One form of inheritance that can give rise to an apparently sporadic condition, especially in small families, is that of recessive variants^4^. Homozygous variants in ALS have already been described in *SOD1*, *OPTN*, and *FUS*^1^, and other rare variants could be responsible to further SALS cases^4^. Recessive inheritance due to rare compound heterozygous variants is another genetic mechanism that can give rise to a sporadic disorder, since both rare variants are unlikely to be reproduced in the next generation. It has often been pointed out that with the demographic shift to smaller families, a disease with a recessive inheritance or with a low penetrance will seem sporadic in a large number of cases^5^.

*De novo* mutation, in which the pathogenetic variant arises for the first time in the offspring of normal parents, is a further mechanism that can give rise to an apparently sporadic disorder. *De novo* mutations in *FUS*^6^, *ERBB4*^7^ and *ATXN2*^8^ have previously been suggested to be associated with ALS.

A powerful method of looking for recessive and *de novo* variants underlying a sporadic disorder is the use of case-unaffected-parents trios. Large numbers of these trios are difficult to collect in ALS, since it is unusual to have access to living parents of ALS patients, with the average age of disease onset being in the early 60 s. In 2011, a genome-wide copy number analysis of 12 SALS trios found a number of *de novo* copy number variants (CNVs) in the SALS offspring; 11 of these CNVs involved genes, some of which were in pathways suspected in the pathogenesis of ALS^9^. More recently, exome sequencing of 47 SALS trios brought to light *de novo* single nucleotide variants in genes that may be involved in the pathogenesis of ALS^10^.

In an attempt to uncover rare recessive and *de novo* variants that could underlie SALS, we therefore sequenced the exomes of 44 Australian case-unaffected-parents trios.

## Results

### ALS offspring patients and unaffected parents

White blood cell DNA samples were available from 44 trios (see Table 1 for the number of recessive and de novo variants found in each ALS patient, and Supplementary Table S1 online for further clinical details of the patients and all ages). Thirty-seven of the offspring had classical sporadic ALS (SALS) with upper and lower motor neuron signs, three had sporadic progressive muscular atrophy (SPMA), two had sporadic progressive bulbar palsy (SPBP), one had sporadic primary lateral sclerosis (SPLS), and one had sporadic frontotemporal degeneration with motor neuron disease (SFTD-MND).

The average age of disease onset of our ALS trio (ALS^TRIO^) offspring was 46.1 y (SD 9.1 y, range 26–63 y). In comparison, the average age of disease onset for the 828 SALS patients in the Australian MND DNA Bank was 61.9 y (SD 11.5 y, range 26–99 y), a significant difference on unpaired two-tailed t-testing (p < 0.0001).

The average age of fathers at the birth of ALS^TRIO^ offspring was 29.4 y (SD 5.1 y, range 22–42 y) and that of the 689 Australian MND Bank fathers at ALS offspring birth was 31.4 y (SD 6.9 y, range 15–67 y), a non-significant difference on t-testing (p = 0.06). The average age of mothers at the birth of ALS^TRIO^ offspring was 26.3 y (SD 4.0 y, range 20–38) and that of the 689 Australian MND Bank mothers at ALS offspring birth was 28.2 y (SD 6.1 y, range 13–50 y), also a non-significant difference on t-testing (p = 0.05).

### Total numbers of variants

The whole exomes of the 44 trios were sequenced to an average of 52.5X coverage (see Methods and Supplementary Methods online for sequencing details). A total of 307,780 variants passed false-positive and site filters, and 305,622 variants passed Hardy-Weinberg Equilibrium tests (p < 0.0001). An average of 55,727 variants per individual was found (which included a 500 bp wingspan from the target space that added an additional 200 kbp of non-coding space with off-target variant calling). Transition/Transversion (Ts/Tv) ratios for coding and non-coding bases per individual (to assess the accuracy of single nucleotide variant filtering), and replacement to silent ratios per individual (to infer the direction and magnitude of natural selection acting on protein coding genes), are available online in Supplementary Tables 2 and 3, respectively.

### Homozygous and compound heterozygote recessive variants

**Figure 1:**
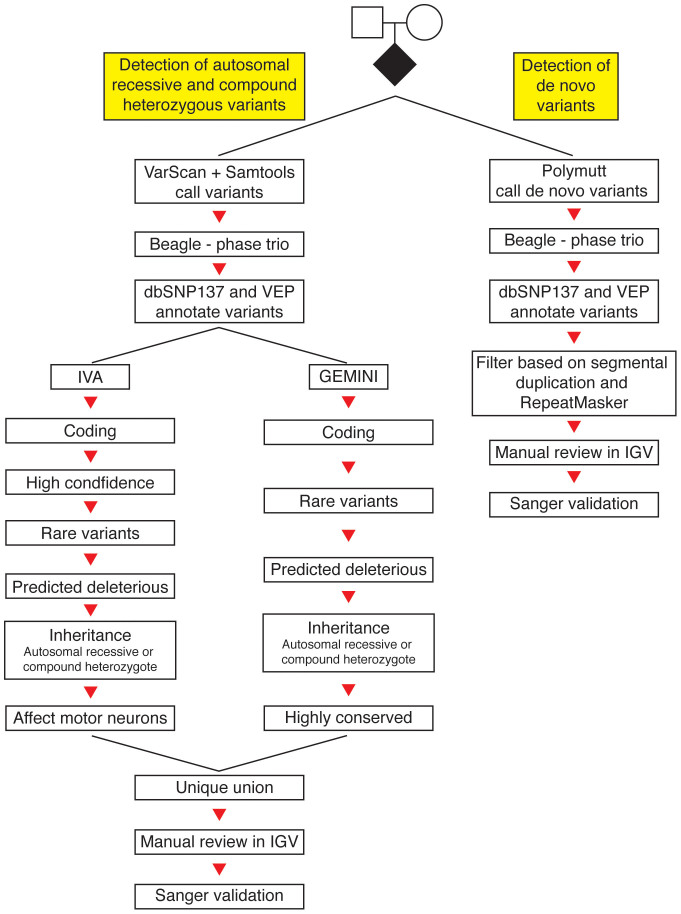
Filtering schema for exome variant calls in case-unaffected parents trios. (1) To identify autosomal recessive and compound heterozygous variants (left track), variants were called using the unique union of VarScan and SAMtools calls. The trio was phased using BEAGLE v4 software and annotated using the NCBI single nucleotide polymorphism database (dbSNP) and Variant Effect Predictor (VEP). Ingenuity variant analysis (IVA) and Genome Mining (GEMINI) software were then used to rigorously filter variants based on quality, minor allele frequency, deleteriousness, inheritance patterns, conservation and involvement in motor neuron pathways (see Supplementary Methods for parameters). The unique union of these variants was manually reviewed and independently validated using Sanger sequencing. (2) To identify *de novo* variants (right track), variants were called using Polymutt, a pedigree-aware variant caller. Phasing and annotation were as per the autosomal recessive and compound heterozygous variants. Variants were then filtered and manually reviewed to eliminate systematic false positives. The remaining variants were validated with Sanger sequencing.

The carrier rate of a potential recessive allele is estimated to be approximately 1% based on an incidence of 2 per 100,000 per year. Out of 16,866 unique autosomal recessive loss of function and nonsynonymous variants, 90 were at global minor allele frequency (MAF) < 1%. Out of 125,006 loss of function and nonsynonymous compound heterozygous, 5,008 were at global MAF < 1%. For these calculations, we required that the transmitted allele in both parents was MAF < 1%. After rigorous filtering based on global MAF, predicted functional consequence, and sequence conservation (for pathways see Figure 1), 49 recessive and compound heterozygous variants remained for validation and further analysis. Full lists of the coding recessive and compound heterozygous variants detected are shown in Supplementary Dataset 1 and Supplementary Dataset 2 respectively online. We validated 28 compound heterozygous variants in 19 different genes (Table 2), which involved 12 (27%) ALS^TRIO^ patients (Table 1). In 6 (14%) ALS^TRIO^ patients 9 homozygous recessive variants in 9 different genes were found (Tables 1 and 2). The deleterious nature of these recessive variants can be judged from their average SIFT score of 0.0058 and average PolyPhen2 score of 0.0008. A quarter of the genes with these recessive variants have significantly increased expression in the spinal cord compared to non-central nervous system tissues^11^ (Table 2).

### *De novo* variants

Eighty-one *de novo* variants passed manual review in IGV and 54 were validated with Sanger sequencing (Figure 1). See Supplementary Table S4 online for the complete list of *de novo* variants. Seventeen of the *de novo* variants were coding, involving 12 (27%) ALS^TRIO^ patients (Tables 1 and 3). Of these 17 variants, 15 were missense (10 identified as deleterious or damaging using SIFT, PolyPhen and Condel), one splice site, and one nonsense. Twenty-four percent of the genes in which we found *de novo* variants have significantly increased expression in the spinal cord compared to non-central nervous system tissues^11^ (Table 3). Although two coding *de novo* variants were found in five ALS^TRIO^ patients, the distribution of *de novo* variants followed a Poisson distribution (see Supplementary Fig. S1 online), indicating that multiple *de novo* alleles in any one individual are unlikely to contribute to ALS risk.

### Relation of variants found to known ALS variants

The frequency of variants in ALS susceptibility genes and the frequency of known ALS susceptibility variants were assessed in our cohort using ALSoD^12^. No increased burden of coding variants in known ALS genes was found in this cohort. All of the coding variants have been previously identified, and the alternate allele frequencies of these variants are similar to those in the NHLBI ESP and the 1000 Genomes Project, with the exception of a few non-synonymous variants that had elevated frequencies (see Supplementary Table S5 for a list of these). Given our limited sample size, however, we were unable to determine whether this enrichment was statistically significant.

### Homozygous segments

No statistically significant enrichment of homozygous segments was found by size, burden, or genomic location in ALS^TRIO^ patients versus controls.

### Functional implications

All except one (*HENMT1*) of the recessive variants were accepted in the DAVID functional annotation analysis. This analysis revealed an enrichment of genes sharing the domain-1 of dynein heavy chain (*DNAH10*, *DNAH2* and *DNAH9*) (p = 0.00006, FDR = 0.004). In addition, the above three genes, along with *ABCA2* and *ATP8B3* (all five genes with ATPase activity and an ATPase-associated domain) were enriched (p = 0.0023, FDR = 0.058). The above five variants also have nucleotide binding capacity, together with *CNGA4*, *MYO3B* and *RAB25* (p = 0.0025, FDR = 0.07). Therefore of these eight genes, all have nucleotide binding capacity, five have ATPase activity, and three have dynein heavy chain domain-1 activity.

Among the 17 genes with *de novo* non-synonymous or splicing variants, *METTL22* was not present in the DAVID identification list and was excluded from the analysis. Within the remaining 16 genes, seven (*LIMD1*, *FOXN3*, *GTF2H4*, *MLL3*, *STK36*, *SND1* and *TRRAP*) are related to regulation of transcription (p < 0.02, FDR < 0.1). Another enriched group, comprising *ANAPC7*, *FOXN3* and *PSMB7*, is involved in cell cycle processes (p < 0.05, FDR < 0.1).

Our DAVID analyses showed no involvement in any functional pathways of genes containing either recessive or *de novo* variants. The previous ALS trio exome study of Chesi et al., on the other hand, which used the same DAVID analysis, reported that chromatin regulator genes were significantly enriched^10^. When we combined our and the Chesi et al. *de novo* variants, and submitted them to functional annotation analysis, genes related to transcription regulation became more significantly enriched than our previous analysis (p = 0.000032, FDR = 0.0018). These 15 enriched genes comprised six from our ALS^TRIO^ list (*LIMD1, FOXN3, GTF2H4, MLL3, SND1* and *TRRAP*) and nine from the Chesi et al. list (*CNOT1, ELL, FOXA1, FOXK1, HDAC10, SRCAP, SS18L1, ZNF410, ZNF778*). This combined analysis therefore gives further weight to the suggestion that disturbances by *de novo* variants of transcription regulation genes may be a pathogenetic mechanism in ALS. On the other hand, genes related to chromatin modification were not significant in the combined *de novo* analysis, with a high false discovery rate (FDR = 0.27).

## Discussion

Due to the late age of onset of ALS, and the possibility of incomplete penetrance, it is difficult to assess whether SALS is truly sporadic. For example, multiple system atrophy was once thought to be sporadic, but recently-identified compound heterozygous and recessive mutations in *COQ2* segregate with the disease, and heterozygous mutations in the same gene predispose individuals to this disease^13^. Additionally, it has been reported that ALS patients harbour a greater number of rare homozygous segments than controls, and that these segments are longer and contain more genes^4^. This suggested further evidence for a recessive cause for apparently sporadic ALS, though our finding of no excess homozygosity in ALS patients does not support this hypothesis of long runs of homozygosity containing rare ALS susceptibility variants. This does not necessarily mean that recessive inheritance can be ruled out, just that long runs of homozygosity were not found in our cohort.

With our dataset of case-parent trios we tested the hypothesis that rare, recessive-acting variants could contribute to disease susceptibility. Indeed, a number of promising candidate genes with recessive or compound heterozygous variants were identified. For example, in one family we identified two extremely rare variants in *ABCA2* that are highly conserved and are predicted to be damaging. *ABCA2* encodes an ATP-binding cassette transporter and plays a role in intracellular sterol trafficking. It is highly expressed in the brain and regulates low-density lipoprotein metabolism in neuronal cells^14^. Dysregulation of *ABCA2* is associated with amyloid beta deposition in Alzheimer's disease^15^, and *ABCA2* null mice accumulate more gangliosides and sphingomyelin in neuronal tissue compared to wild-type mice^16^.

We identified a recessive variant in *RAB25* in one ALS^TRIO^ patient. This gene encodes a protein involved in membrane trafficking and has nucleotide binding capacity. A meta-analysis of genome-wide association studies showed that a common variant at the *SYT11*/*RAB25* locus is associated with Parkinson's disease in Caucasians^17^, suggesting a role for this gene in neurodegenerative diseases.

*CACNA1H* encodes a protein in the voltage-dependent calcium channel complex, and we identified two extremely rare damaging variants inherited as a compound heterozygote in one ALS^TRIO^ patient. Dysregulation of calcium homeostasis in spinal and motor neurons has been previously demonstrated in mouse models of ALS^18^. This leads to altered excitability of motor neurons with modified synaptic activity and neuronal excitotoxicity^19^. Of interest, in presymptomatic ALS patients cortical hyperexcitability appears to be an early feature^20^. Our results give more weight to the idea that variants in voltage-dependent calcium channel genes play a role in ALS susceptibility.

Functional annotation analysis of the recessive variants showed enrichment for genes that are involved in the dynein heavy chain (*DNAH10*, *DNAH2* and *DNAH9*). This is of interest since defects in axonal transport have long been suspected to play a part in ALS^21^. A group of five genes, comprising the three dynein-related genes above, as well as *ABCA2* and *ATP8B3,* were enriched for ATPase activity. Na,K-ATPase has been suggested to be involved in mutant-*SOD1* ALS^22^, but data on the activity of other forms of APTase in ALS are sparse, despite the fact that altered energy metabolism is a possible mechanism in ALS^23^. Finally, the above five genes, as well as a further three (*CNGA4*, *MYO3B* and *RAB25*) are enriched for nucleotide binding activity. Of note, caution needs to be exercised in attributing importance to the variants in *DNAH10* and *MYO3B* since exome sequencing frequently finds variants in these genes^24^.

Recent studies of individual SALS patients and their parents have identified *de novo* variants in ALS-associated genes such as *FUS*^25^ and *CREST*^26^. Other sporadic disorders such as autism spectrum disorder demonstrate a similar pattern of recurrent *de novo* variants^27^. Interestingly, we identified a novel *de novo* initiator codon variant in *CHRM1*, a gene that also harbored a *de novo* missense variant in a previous ALS exome trio study^10^. This gene encodes a cholinergic receptor and is predicted to be involved in diseases of motor neurons and frontotemporal dementia, which is related to ALS. *CHRM1* is predominantly expressed in the parasympathetic nervous system and influences the effects of acetylcholine in the central and peripheral nervous systems. In patients with Alzheimer's disease, loss of *CHRM1* exacerbates cognitive decline^28^ and increases amyloid pathology^29^. In spinal cord injuries significantly reduced gene expression of muscarinic cholinergic receptors intensifies motor dysfunction^30^. Our results further support the hypothesis that damaging variants in *CHRM1* contribute to neurodegenerative disorders such as ALS.

Of note, *CHRM1* was the only gene in which *de novo* variants (in different regions of the gene) were found in both our and the previous ALS trio exome study of Chesi et al.^10^, with the two studies containing a total of 91 ALS patients. This implies that, if *de novo* mutations do play a major part in ALS, large numbers of private mutations in different genes are likely to be responsible for the disease.

We identified a novel coding *de novo* variant in *ITPR2*. Common variations in this gene have been associated with ALS, with the expression of *ITPR2* being increased in the peripheral blood of ALS patients^31^. *ITPR2* is highly expressed in motor neurons where it encodes a calcium channel on the endoplasmic reticulum, the latter a site a great interest in ALS^32^. Dysfunction of *ITPR2* with increased intracellular calcium may lead to motor neuron cell death^31^ and overexpression of murine *ITPR2* in the *SOD1*^G93A^ ALS mouse model damages cells by increasing the release of neuronal calcium^33^. In neuronal cell lines, oxidative stress leads to calcium dysregulation by upregulating *ITPR2* expression, which increases calcium release into the nucleus^34^. The association of *ITPR2* with ALS has not been replicated in other genome-wide association^35^ or single nucleotide variant studies, though these only assayed common, and not rare, variants. Our results, on the other hand, suggest that rare variants in this gene may contribute to the pathogenesis of ALS.

Functional annotation analysis of our *de novo* variants showed seven that are related to transcription regulation (*LIMD1*, *FOXN3*, *GTF2H4*, *MLL3*, *STK36*, *SND1* and *TRRAP*), which is in keeping with the findings of abnormal RNA transcription and processing in ALS^36^. Caution though needs to be exercised attributing significance to the *de novo* variant found in *STK36* since this gene frequently contains variants in exome sequencing^24^. The finding of *de novo* variants in three genes related to the cell cycle (*ANAPC7*, *FOXN3* and *PSMB7*) is in accord with suggestions that cell cycle abnormalities underlie some instances of ALS^37^.

It has been suggested that ALS may be caused by variants in a number of genes within one individual, the so-called oligogenic hypothesis^38^. Our findings support this hypothesis, since nine ALS^TRIO^ patients had more than one gene with either a recessive or *de novo* rare variant. The Poisson distribution for novel *de novo* coding variants in our study suggests that these variants alone are unlikely to be involved in an oligogenic process. However, 75% of our ALS patients who had *de novo* variants had concurrent recessive or other *de novo* variants; only 3 patients had a single *de novo* variant, and in these it is quite possible that other recessive or *de novo* variants outside of the exome sequencing targets could play contributory roles.

Although evidence for an oligogenic mechanism for ALS was present in our present study, we looked only at single nucleotide variants. Other genetic abnormalities, such as copy number variants, DNA methylation^39^, or somatic mutations^40^ could interact with the variants we found in our ALS^TRIO^ patients to confer further susceptibility to disease. For example, when 12 of the present ALS^TRIO^ patients had genome-wide CNVs analysed with microarrays in a previous study^40^, *de novo* CNVs were found in 11 of them (Table 1). CNVs that overlapped with genes or promoters were found in eight of these patients, including three with multiple CNVs.

ALS trio studies are uncommon, and without access to parent DNA we do not know how many mutation-carrying parents of ALS patients never develop the disease, or develop it at a much older age than their offspring. ALS-associated variants were found in our study in four ALS^TRIO^ patients as well as in an unaffected parent; one of these was in *SOD1*, two in *C9orf72*, and one in *TDP-43*^41^. Either environmental or modifying genetic variations could be responsible for this difference in phenotype between parent and offspring. Unaffected mutation-bearing parents could also carry a protective genetic variant elsewhere in their genomes. Our finding that all four of the above ALS^TRIO^ patients had additional single nucleotide or copy number variants suggests that other genetic variants may be needed for the ALS phenotype to appear in some patients who have apparently single gene mutations.

Limitations of the present study are: (1) Our ALS patients had a younger average age of onset that is usual for this disease, so they could represent a different subgroup where genetic variants are more common than in most sporadic ALS. (2) A parent could present with the onset of ALS much later in life (a not uncommon clinical scenario), so we cannot be sure that the ALS in our trios was truly of an isolated/sporadic nature. (3) We did not analyse the whole genome, so potentially significant recessive or *de novo* variants in intronic or intergenic regions could not be detected. (4) Further assessment of compound heterozygote and *de novo* variant frequency in ALS will only be able to be undertaken once larger numbers of ALS trios become available, which will require an international collaborative effort. A number of groups are presently undertaking exome sequencing on large numbers of individual ALS patients, so whether the variants we found are truly private mutations or are more common will soon be known. Not having parental DNA, however, means these studies will not be able to determine whether the variants are actually recessive or *de novo* in nature. (5) Exome capture is inherently biased towards the creation of false positives. For this reason we imposed several quality control steps in an attempt to filter out false positives. For the *de novo* variants we used Polymutt software that takes into account the parental genotypes when calling a *de novo* variant in the offspring. Of the variants that did not validate, 14 were due to poor Sanger data quality in one or both of the parents and four were due to poor Sanger data quality in the offspring. Seven variants were actually homozygous reference in the child and two variants were present (but undercalled from exome data) in the parents, representing true false positives. Although there are slightly more *de novo* variants than would be expected from a true exome (<1 per family), most of these were non-coding or silent mutations. There were only 17 coding (15 missense) *de novo* variants out of the 44 families; this number is in line with *de novo* coding events in other exome studies^42^. (6) Because of our relatively small number of trios, we did not have sufficient numbers to undertake a rare variant transmission disequilibrium test (TDT) that would yield adequate statistical power^43^,^44^.

As is common in other genomics-based studies, we expect that the variants we found will be a springboard for other researchers to develop model systems to further explore their functionality. We consider all our 28 recessive and 17 *de novo* variants to be strong candidates for a role in ALS, since our vigorous *in silico* analyses ensured that we reported only validated variants that are rare and involved in processes or metabolic pathways implicated in ALS. Complex model systems will be needed to test the functionality of these variants, since testing has to take into account the probability that multiple variants are acting together, and that exposure to environmental toxins, such as heavy metals^45^ and neurotoxic amino acids^46^, are also playing a part in the disease. Future studies using a combination of whole genome nucleotide sequences, structural variations, and epigenetic differences, using multiple tissues to look for somatic mutations, and obtaining DNA from multiple generations, are likely to be needed to uncover all the variants comprising the genetic contribution to sporadic ALS.

In conclusion, our exome sequencing of ALS-unaffected-parents trios has uncovered rare homozygous, compound heterozygous, and *de novo* variants that are likely to play a role in the pathogenesis of this disease. Most of these appear to be private variations, which implies that we will be unlikely to find any more mutations (such as those in *C9orf72*) that are common to large numbers of sporadic ALS patients. The implications of this study are four-fold: firstly, there are no previously published ALS trio exome studies showing the widespread occurrence of potentially deleterious compound heterozygous variants. Secondly, only one previous ALS trio study has demonstrated *de novo* variants, and our study confirmed these do occur, though in different genes (apart from one shared between the two studies), indicating that most are likely to be rare or private variants. Thirdly, we validated extremely rare, highly conserved, deleterious recessive mutations in our sporadic ALS patients. Hidden recessive inheritance in ALS has been hypothesised for many years, and we have now been able to show the importance of this mode of inheritance that could explain the sporadic nature of some ALS, possibly in combination with other recessive or *de novo* variants. Finally, our findings give the best evidence so far that oligogenic variants underlie much of sporadic ALS.

## Methods

### Ethics statement

The study protocol was approved by the Sydney South West Area Health Service Human Research Ethics Committee. Informed written consent was obtained from each individual for their DNA to be used for research purposes. All methods were carried out in accordance with the approved guidelines and regulations.

### SALS patients and unaffected parents

Individuals selected for study were patients with ALS who had donated blood samples to the Australian Motor Neuron Disease DNA Bank, and whose ALS-unaffected parents had also given blood samples to the Bank. The diagnosis of ALS was made by a neurologist using standard criteria. For the purpose of this study, patients were considered to have “sporadic” ALS if they had no history of ALS in any family member at the time of blood sampling, even if an ALS-associated mutation was found in that patient and their family member. All ALS offspring in this study are referred to as “ALS Trio” (ALS^TRIO^) patients.

### Exome sequencing

For details of exome sequencing see Supplementary Methods online, and for sequencing metrics see Supplementary Table S6 online.

### Variant calling and annotation pipelines

Figure 1 outlines the methods used to filter the exome variant calls to detect autosomal recessive, compound heterozygous, and *de novo* variants in the ALS offspring of the trios. For details of variant calling and annotation pipelines see Supplementary Methods online.

### Validation of variants

For details of the method to validate the variants see Supplementary Methods online.

### ALSoD analysis

Data from the ALS online database (http://alsod.iop.kcl.ac.uk/) were downloaded, and variant calls from the exomes were intersected with the previously identified ALS susceptibility variants. The affected and unaffected carrier frequencies were calculated using GEMINI, a framework for exploring genome variation.

### Homozygous segments

Thirty of 44 ALS probands were genotyped on the Illumina Human Omni Express Bead Chip. Control data were drawn from the 379 European descent 1000 Genomes individuals that were genotyped on the Illumina Omni 2.5 Bead Chip. Data for both sets were imported into the whole genome analysis tool PLINK (v.1.07)^47^ and standard quality control procedures were applied. Samples were excluded if they had call rates <95%, single nucleotide polymorphisms (SNPs) with minor allele frequencies < 0.01, Hardy-Weinberg equilibrium p-values < 0.0001, or non-random missingness in cases versus controls. The two datasets were combined and the intersection of the two marker lists was used for a total of 667,708 SNPs genome-wide. SNPs were pruned based on linkage disequilibrium using a “light” pruning scheme^48^, where SNPs with r^2^ > 0.9 in a 50 SNP window were removed, leaving 307,288 SNPs. In addition, none of the cases were outliers from the 1000 Genomes European ancestry populations based on PLINK multidimensional scaling analysis. Runs of homozygosity (segments >2 Mb) were identified from the autosomal chromosomes in PLINK^4^ and burden and association analyses were performed as previously described^4^, with the exception that the gene list was taken from the UCSC Table Browser hg19 RefSeq genes. Briefly, homozygous segments were coded as copy number variants and analyzed using the PLINK rare copy number variant burden and association analysis. p-values were generated from 100,000 case/control permutations and statistical significance was set at a genome-wide corrected p-value < 0.05.

### Functional implications

To predict the functional implications of the identified variants, lists of *de novo* and recessive variants were generated from the exome sequencing data and submitted to the Database for Annotation, Visualization and Integrated Discovery (DAVID 6.7)^49^. The NimbleGen SeqCap EZ Human Exome gene list was used as a background. Potential functional enrichments and pathway analysis were explored, with p-values < 0.05 and false discovery rates < 0.1 selected as significant. Pathway analysis was also undertaken on the combined *de novo* variant findings in our and in the Chesi et al. ALS trio exome study^10^.

## Author Contributions

K.M.S. performed the variant calling, analysed the exome sequencing data with IVA and GEMINI, performed the ALSoD and homozygosity analyses and co-drafted the article. B.Y. performed functional annotation analyses and assisted in data analysis and writing the article. D.K. assisted in data analysis and writing the article. E.M. contributed to study design, writing the article and gave final approval for publication of Washington University School of Medicine data. R.P. conceived the study, supplied DNA samples and clinical information, contributed to the study design, co-drafted the article, and gave final approval for publication of University of Sydney data.

## Additional Information

**Accession codes:** All appropriate datasets are available in the database of Genotypes and Phenotypes (dbGaP) (accession number phs000831).

## Supplementary Material

Supplementary Information Supplementary Information

Supplementary Information Dataset 1

Supplementary Information Dataset 2

## Figures and Tables

**Table 1:** Clinical details and numbers of recessive and *de novo* variants detected in ALS trio patients

Trio ID	Gender	Diagnosis	Age at onset	Homozygous variants	Compound heterozygous variants	De novo variants	De novo CNVs (previous study)	ALS mutation
#01	Male	ALS	44					
#02	Female	ALS	49			2	2	
#03	Female	ALS	35				3	
#04	Female	ALS	26				4	
#05	Female	ALS	50		1		4	
#06	Male	ALS	36				3	C9orf72 F
#07	Male	ALS	36				3	
#08	Female	PMA	51			2	3	
#09	Male	ALS	53				4	
#10	Male	ALS	44			1	3	
#11	Female	ALS	53		1		3	
#12	Female	PBP	58	1			4	
#13	Male	ALS	53	1			na	
#14	Male	ALS	27		2	1	na	
#15	Male	ALS	35		1		na	SOD1 F
#16	Male	FTDMND	58		1		na	
#17	Male	ALS	51				na	
#18	Female	ALS	47				na	
#19	Male	ALS	53		1		na	TDP-43 M
#20	Male	ALS	56	4		1	na	
#21	Male	ALS	45			1	na	
#22	Male	ALS	46	1			na	
#23	Male	ALS	44		1		na	
#24	Male	ALS	36		2	2	na	
#25	Female	ALS	42				na	
#26	Male	ALS	41		1		na	
#27	Male	ALS	47				na	
#28	Male	PMA	55				na	
#29	Female	PBP	55				na	
#30	Male	ALS	50	1		2	na	
#31	Female	ALS	53				na	
#32	Male	ALS	57				na	
#33	Male	ALS	42				na	
#34	Male	ALS	48				na	
#35	Male	ALS	46			2	na	C9orf72 F
#36	Male	ALS	59		1		na	
#37	Male	ALS	28				na	
#38	Female	ALS	45				na	
#39	Female	ALS	53	1			na	
#40	Female	ALS	63				na	
#41	Male	ALS	37		1	1	na	
#42	Male	ALS	30		1	1	na	
#43	Female	PLS	45			1	na	
#44	Male	ALS	46				na	

The ALS trio patients had a younger average age than usual for ALS. Most patients had classical ALS. Included in the table are *de novo* CNVs from 12 of the patients that were found in a previous study^9^. Four ALS trio patients who had known ALS-associated mutations also had recessive or *de novo* variants. CNV: copy number variant. F: mutation also found in father, M: mutation also found in mother, na: not applicable.

**Table 2:** Recessive homozygous or compound heterozygous variants in ALS trio patients

Gene	Trio ID	Chrom	Position	Recessive inheritance	Impact	Amino acid change	Minor allele frequency ESP	Minor allele frequency 1KG	dbSNP 137 rsID	Spinal cord differential expression
ABCA2	#24	9	139908464	Cpd Het	Missense	V1422F	0.008303	0.01	rs147917446	Up***
ABCA2	#24	9	139916347	Cpd Het	Missense	M224K	NR	NR	NR	Up****
ATP8B3	#16	19	1788909	Cpd Het	Missense	G1019D	0.003602	0	rs202137046	Down*
ATP8B3	#16	19	1796751	Cpd Het	Missense	C524Y	NR	NR	NR	Down*
CACNA1H	#14	16	1265267	Cpd Het	Missense	V1683M	NR	NR	NR	Down**
CACNA1H	#14	16	1265315	Cpd Het	Missense	A1699T	0.006668	0.0041	rs148651456	Down**
CNGA4	#24	11	6261613	Cpd Het	Missense	G197R	NR	NR	NR	ND
CNGA4	#24	11	6261718	Cpd Het	Missense	V232M	NR	NR	NR	ND
DENND2C	#20	1	115130508	Homozygous	Missense	Y833H; Y776H	0.004075	0.0023	rs61753528	Down**
DNAH10	#23	12	124323006	Cpd Het	Missense	M1518V	0.009286	0.0027	rs145483216	Up**
DNAH10	#23	12	124409693	Cpd Het	Missense	R3837C	0.002361	0.0027	rs144421774	Up**
DNAH2	#41	17	7727209	Cpd Het	Missense	R3757H	NR	NR	NR	ND
DNAH2	#41	17	7734055	Cpd Het	Missense	G4003V	NR	NR	NR	ND
DNAH9	#26	17	11775004	Cpd Het	Missense	L1963F	NR	NR	NR	ND
DNAH9	#26	17	11840674	Cpd Het	Missense	I2671M	0.001307	NR	rs143953217	ND
EIF4E1B	#20	5	176072210	Homozygous	Missense	R147H	0.003643	0.0023	rs115365515	Up****
GORASP1	#19	3	39140352	Cpd Het	Missense	D162Y	0.00692	0.01	rs13886448	Up***
GORASP1	#19	3	39142562	Cpd Het	Missense	A127V	0.004306	0.0037	rs61743223	Up***
GTF3C2	#20	2	27558834	Homozygous	Missense	L473V	0.002537	0.0005	rs148867164	Down*
HENMT1	#13	1	109193733	Homozygous	Missense	E166A	0.000461	0.0009	rs144705350	Up**
KIAA1755	#12	20	36848055	Homozygous	Missense	R845C	0.000384	NR	rs144671254	Up*
LBP	#14	20	36978016	Cpd Het	Missense	G64R	NR	NR	NR	ND
LBP	#14	20	36979309	Cpd Het	Missense	V112D	0.000538	0.0005	rs138570528	ND
MYO3B	#42	2	171356232	Cpd Het	Missense	Q1067R	0.000248	0.0005	rs200292179	ND
MYO3B	#42	2	171400401	Cpd Het	Splice site	None	NR	NR	NR	ND
RAB25	#20	1	156035717	Homozygous	Missense	E20G	0.005891	0.01	rs61751627	Down****
SERPINA10	#15	14	94750486	Cpd Het	Missense	Q384R	0.008304	0.0037	rs2232710	Down*
SERPINA10	#15	14	94756669	Cpd Het	Nonsense	R88X	0.005305	0.0032	rs2232698	Down*
SPTB	#11	14	65253667	Cpd Het	Missense	A1006T	0.001153	NR	rs151112486	ND
SPTB	#11	14	65267517	Cpd Het	Missense	S278F	NR	NR	NR	ND
TAF1L	#39	9	32631781	Homozygous	Missense	P1266R	0.002076	NR	rs140558556	NR
TF	#30	3	133496032	Homozygous	Missense	G671E	0.003691	0.0032	rs121918677	Up****
THSD7B	#22	2	138373831	Homozygous	Missense	Q1141H	0.004745	0.0009	rs150657202	ND
USH2A	#05	1	215844468	Cpd Het	Missense	P4660L	NR	NR	NR	ND
USH2A	#05	1	216420214	Cpd Het	Missense	S841Y	0.005305	0.0027	rs111033282	ND
WDR6	#36	3	49049385	Cpd Het	Missense	L140V	NR	NR	NR	ND
WDR6	#36	3	49050499	Cpd Het	Missense	R460H	0.000846	0.0009	rs142520902	ND

The minor allele frequencies from the NHLBI Exome Sequencing Project (ESP) and 1000 Genomes Project (1KG) projects and the dbSNP137 rsID are provided. Differential expression of each gene in the spinal cord compared to non-central nervous system tissue from^11^ is noted. Cpd Het: compound heterozygous. ND: no difference, NR: not reported,*: p < 10^−2^, **: p < 10^−4^, ***: p < 10^−6^, ****: p < 10^−10^.

**Table 3:** Rare coding *de novo* variants in ALS trio patients

Gene	Trio ID	Chrom	Position	Impact	Amino acid change	Minor allele frequency in ESP	Minor allele frequency in 1KG	dbSNP137 ID	Novel	Spinal cord differential expression
AKD1	#08	6	109894726	Missense	E755K	NR	NR	NR	Yes	Up**
ANAPC7	#21	12	110819574	Missense	R108H	NR	NR	NR	Yes	Down**
CHRM1	#41	11	62678572	Missense, initiator codon	M1V	NR	NR	NR	Yes	Down*
FOXN3	#30	14	89656737	Nonsense	Q119X	NR	NR	NR	Yes	Down*
GTF2H4	#35	6	30880156	Missense	R337Q	0.010308	0.01	rs3218820	No	NR
ITPR2	#42	12	26808680	Missense	F850L	NR	NR	NR	Yes	Up****
LIMD1	#02	3	45637047	Missense	P226S	NR	NR	NR	Yes	Down**
METTL22	#30	16	8738455	Missense	A295V	NR	NR	NR	Yes	Down**
MLL3	#02	7	151849993	Missense	R234Q	NR	NR	NR	Yes	Down*
NLRC5	#43	16	57073761	Missense, splice site	R256M	NR	NR	NR	Yes	Down**
PLA2G4C	#24	19	48607867	Missense	A79S	NR	NR	rs13895674	No	Up***
PSMB7	#08	9	127119118	Missense	I216T	NR	NR	NR	Yes	Down***
RINL	#10	19	39359972	Missense	R404Q	NR	NR	NR	Yes	NR
SND1	#24	7	127341354	Missense	S179L	NR	NR	rs24667910	No	ND
STK36	#14	2	219538460	Missense	M12R	NR	NR	NR	Yes	Down*
SV2A	#20	1	149885128	Missense	E89K	NR	NR	NR	Yes	Up****
TRRAP	#35	7	98553842	Missense	S1977N	NR	NR	NR	Yes	ND

The minor allele frequencies from the NHLBI Exome Sequencing Project (ESP) and 1000 Genomes Project (1 KG) projects and the dbSNP137 rsID are provided. Differential expression of each gene in the spinal cord compared to non-central nervous system tissue from^11^ is noted. ND: no difference, NR: not reported,*: p < 10^−2^, **: p < 10^−4^, ***: p < 10^−6^, ****: p < 10^−10^.
