# Supplementary material for: Exome sequencing of case-unaffected-parents trios reveals recessive and de novo genetic variants in sporadic ALS
Source: Sci Rep. 2015 Mar 16;5:9124. doi: 10.1038/srep09124 (PMC4360641; doi:10.1038/srep09124)
Supplement: Supplementary Information [file srep09124-s1.pdf]

SUPPLEMENTARY INFORMATION

Exome sequencing of case-unaffected-parents trios reveals recessive and *de novo* genetic variants in sporadic ALS

Karyn Meltz Steinberg, Bing Yu, Daniel C Koboldt, Elaine R Mardis, Roger Pamphlett

Supplementary Information: Titles and Captions

Supplementary Figure S1. Frequency distribution of novel non-synonymous *de novo* variants in ALS^{TRIO} patients. The red line indicates the Poisson distribution (p = 0.5). The data fit well with the Poisson distribution suggesting that ALS risk is not caused by multiple *de novo* variants in a single individual.

Supplementary Table S1. Ages and clinical details of ALS^{TRIO} patients, and ages of their parents. Blood sampling took place shortly after the onset of ALS in most patients. Weakness started in the limbs in most patients. The average ages of both fathers and mothers at the time of the ALS^{TRIO} patients' births were similar to a large population of ALS parents in the Australian MND DNA Bank.

Supplementary Table S2. Transition transversion ratio (Ts/Tv) for coding, non-coding, and all bases per individual. Ts:Tv is the transition to transversion ratio. Across the whole genome, transitions (A>G and C>T) are expected to occur twice as frequently as transversions (A>C, A>T, G>C, G>T). In protein coding regions, the ratio observed is often slightly above 3. These values are comparable with previous whole exome sequencing studies.

Supplementary Table S3. Replacement (amino acid change) to silent (no amino acid change) ratio per individual. If a locus is evolving under neutral selection (i.e. no positive or negative selection) the ratio of replacement to silent sites should be constant. We tested for replacement to silent ratios greater than or less than one to identify sites that were under stronger or weaker selection. A simple calculation of the number of replacement divided by the number of silent sites gives an estimate of the rate of replacement to silent substitution. We found an enrichment of replacement sites (most likely caused by the annotation software) that chose the most damaging annotation when multiple annotations were available. Alt: alternative allele, Het: heterozygous, Hom: homozygous, Rep: replacement, Sil: silent.

Supplementary Table S4. All coding and non-coding rare *de novo* variants detected in ALS trio patients. The 54 *de novo* variants identified and validated are listed. The minor allele frequencies from the NHLBI ESP and 1KG projects and the dbSNP137 rsID are provided. Most variants are not previously reported and the previously reported variants that are in dbSNP137 have global minor allele frequencies less than 1%. Common variants that underwent Sanger validation passed manual review in IGV as *de novo* variants.

Supplementary Table S5. Coding variants in ALSoD genes in ALS trio patients. ALS coding susceptibility variants from ALSoD were assessed for frequency in this cohort. The global minor allele frequencies of each variant are shown in the NHLBI Exome Sequencing Project (ESP), 1000 Genomes Project (1KG), unaffected carriers in the present study, and affected carriers in the present study. Most variants are common alleles, and the global MAF of the alleles is similar to the frequencies in unaffected and affected carriers in this cohort.

Supplementary Table S6. Sequencing metrics. These values show the mapping and coverage information for the exome sequencing. The majority of reads aligned with a low rate of duplicate reads. We achieved almost 90% of bases mapping on target at 20X coverage. The transition transversion ratio (Ts:Tv) for coding bases is comparable with previous whole exome sequencing studies.

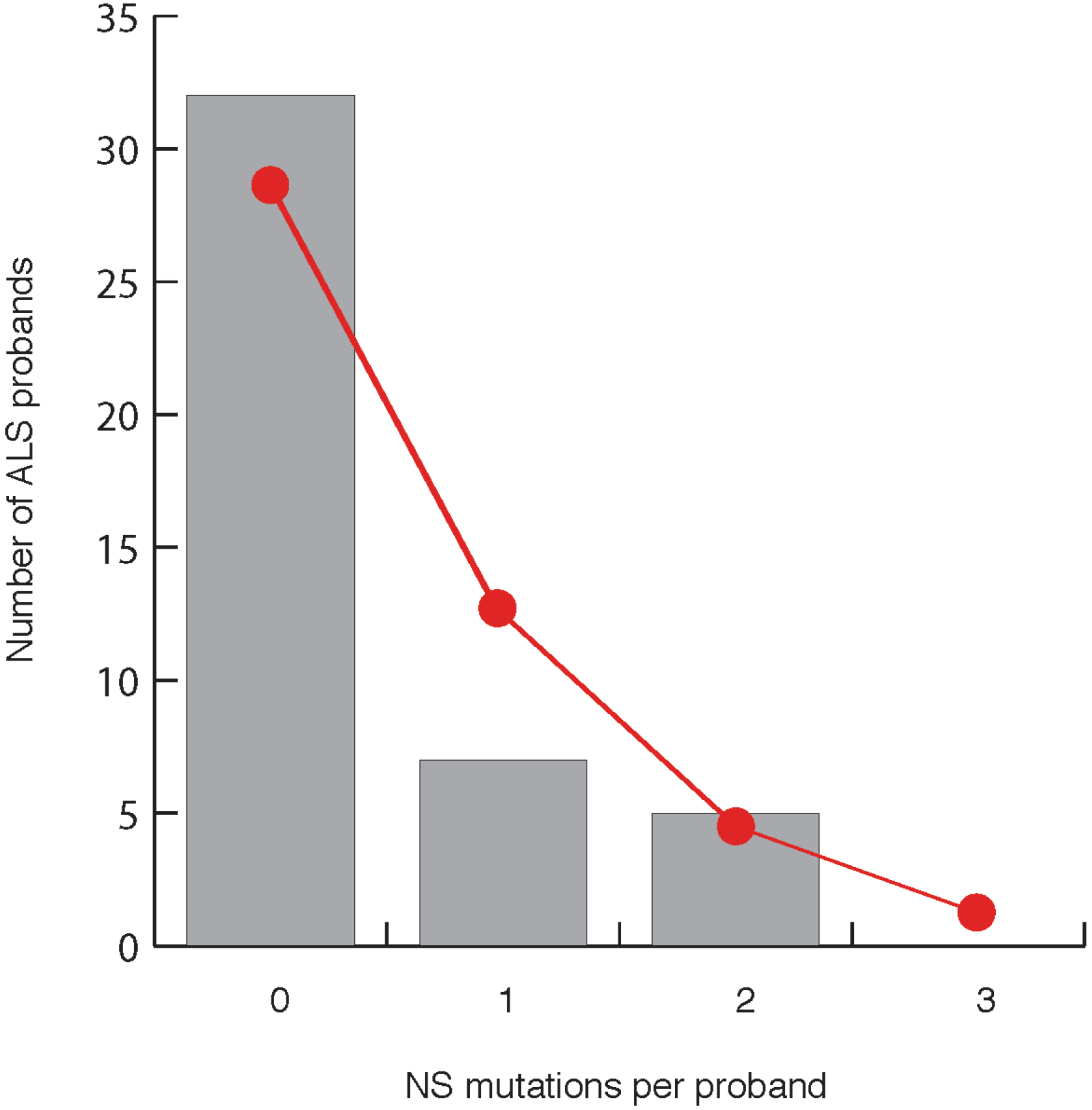
Number of ALS probands
35
30
25
20
15
10
5
0
0
1
2
3
NS mutations per proband

Supplementary Table S1: Ages and clinical details of ALS trio patients, and ages of their parents

Trio ID	Gender	Diagnosis	Age at blood sampling	Part affected first	Father's age at blood sampling	Father's age at ALS birth	Mother's age at blood sampling	Mother's age at ALS birth
Trio#01 ALS	Male	ALS	45	Lower limb	72	27	72	27
Trio#02 ALS	Female	ALS	49	Bulbar	80	31	75	26
Trio#03 ALS	Female	ALS	35	Upper limb	65	30	59	24
Trio#04 ALS	Female	ALS	27	Upper limb	58	31	54	27
Trio#05 ALS	Female	ALS	50	Bulbar	81	31	79	29
Trio#06 ALS	Male	ALS	36	Upper limb	66	30	62	26
Trio#07 ALS	Male	ALS	37	Upper limb	64	27	62	25
Trio#08 ALS	Female	PMA	53	Upper limb	81	28	83	30
Trio#09 ALS	Male	ALS	54	Upper limb	82	28	78	24
Trio#10 ALS	Male	ALS	45	Upper limb	80	35	79	34
Trio#11 ALS	Female	ALS	55	Lower limb	80	25	79	24
Trio#12 ALS	Female	PBP	58	Bulbar	84	26	80	22
Trio#13 ALS	Male	ALS	53	Lower limb	81	28	75	22
Trio#14 ALS	Male	ALS	39	Lower limb	66	27	63	24
Trio#15 ALS	Male	ALS	37	Upper limb	67	30	65	28
Trio#16 ALS	Male	FTDMND	59	Upper limb	86	27	83	24
Trio#17 ALS	Male	ALS	52	Bulbar	80	28	76	24
Trio#18 ALS	Female	ALS	50	Bulbar	78	28	72	22
Trio#19 ALS	Male	ALS	56	Upper limb	86	30	80	24
Trio#20 ALS	Male	ALS	57	Bulbar	82	25	80	23
Trio#21 ALS	Male	ALS	45	Bulbar	72	27	73	28
Trio#22 ALS	Male	ALS	47	Lower limb	88	41	85	38
Trio#23 ALS	Male	ALS	45	Upper limb	74	29	72	27
Trio#24 ALS	Male	ALS	37	Upper limb	76	39	63	26

Trio#25 ALS	Female	ALS	45	Upper limb	70	25	71	26
Trio#26 ALS	Male	ALS	44	Upper limb	70	26	74	30
Trio#27 ALS	Male	ALS	48	Upper limb	78	30	71	23
Trio#28 ALS	Male	PMA	56	Lower limb	80	24	82	26
Trio#29 ALS	Female	PBP	56	Bulbar	79	23	82	26
Trio#30 ALS	Male	ALS	50	Upper limb	91	41	82	32
Trio#31 ALS	Female	ALS	55	Lower limb	80	25	76	21
Trio#32 ALS	Male	ALS	57	Bulbar	80	23	77	20
Trio#33 ALS	Male	ALS	43	Bulbar	67	24	67	24
Trio#34 ALS	Male	ALS	49	Upper limb	87	38	83	34
Trio#35 ALS	Male	ALS	47	Bulbar	73	26	72	25
Trio#36 ALS	Male	ALS	59	Upper limb	92	33	90	31
Trio#37 ALS	Male	ALS	30	Bulbar	59	29	56	26
Trio#38 ALS	Female	ALS	47	Lower limb	89	42	82	35
Trio#39 ALS	Female	ALS	54	Upper limb	79	25	77	23
Trio#40 ALS	Female	ALS	63	Bulbar	85	22	85	22
Trio#41 ALS	Male	ALS	38	Bulbar	75	37	70	32
Trio#42 ALS	Male	ALS	34	Upper limb	64	30	60	26
Trio#43 ALS	Female	PLS	53	Lower limb	89	36	77	24
Trio#44 ALS	Male	ALS	50	Upper limb	75	25	75	25

Supplementary Table S2: Transition transversion ratio (Ts:Tv) for coding, non-coding, and all bases per individual

Trio ID	Coding Ts/Tv	Non-coding Ts/Tv	All Ts/Tv
Trio#01 ALS	3.323	2.318	2.547
Trio#01 Father	3.292	2.325	2.545
Trio#01 Mother	3.297	2.347	2.567
Trio#02 ALS	3.283	2.360	2.574
Trio#02 Father	3.290	2.333	2.558
Trio#02 Mother	3.333	2.354	2.579
Trio#03 ALS	3.335	2.365	2.589
Trio#03 Father	3.270	2.342	2.559
Trio#03 Mother	3.278	2.349	2.566
Trio#04 ALS	3.252	2.341	2.554
Trio#04 Father	3.286	2.337	2.562
Trio#04 Mother	3.223	2.344	2.547
Trio#05 ALS	3.345	2.332	2.564
Trio#05 Father	3.415	2.364	2.608
Trio#05 Mother	3.274	2.347	2.566
Trio#06 ALS	3.286	2.338	2.559
Trio#06 Father	3.265	2.340	2.557
Trio#06 Mother	3.323	2.339	2.566
Trio#07 ALS	3.371	2.340	2.576
Trio#07 Father	3.333	2.351	2.582
Trio#07 Mother	3.314	2.334	2.561
Trio#08 ALS	3.315	2.330	2.557
Trio#08 Father	3.358	2.343	2.576
Trio#08 Mother	3.289	2.358	2.581
Trio#09 ALS	3.335	2.362	2.595
Trio#09 Father	3.391	2.356	2.600
Trio#09 Mother	3.320	2.347	2.584
Trio#10 ALS	3.267	2.358	2.579
Trio#10 Father	3.310	2.360	2.579
Trio#10 Mother	3.217	2.352	2.557
Trio#11 ALS	3.290	2.362	2.584
Trio#11 Father	3.240	2.340	2.552
Trio#11 Mother	3.270	2.339	2.559
Trio#12 ALS	3.282	2.340	2.562
Trio#12 Father	3.293	2.340	2.563
Trio#12 Mother	3.228	2.353	2.559
Trio#13 ALS	3.357	2.346	2.586
Trio#13 Father	3.332	2.343	2.576

Trio#13 Mother	3.261	2.359	2.577
Trio#14 ALS	3.284	2.335	2.558
Trio#14 Father	3.223	2.330	2.540
Trio#14 Mother	3.274	2.357	2.574
Trio#15 ALS	3.266	2.339	2.556
Trio#15 Father	3.318	2.355	2.580
Trio#15 Mother	3.274	2.343	2.568
Trio#16 ALS	3.303	2.325	2.547
Trio#16 Father	3.281	2.324	2.548
Trio#16 Mother	3.288	2.341	2.567
Trio#17 ALS	3.234	2.345	2.555
Trio#17 Father	3.275	2.336	2.555
Trio#17 Mother	3.264	2.327	2.545
Trio#18 ALS	3.310	2.324	2.552
Trio#18 Father	3.317	2.338	2.567
Trio#18 Mother	3.292	2.326	2.549
Trio#19 ALS	3.301	2.344	2.564
Trio#19 Father	3.315	2.363	2.580
Trio#19 Mother	3.357	2.321	2.560
Trio#20 ALS	3.425	2.317	2.574
Trio#20 Father	3.381	2.348	2.599
Trio#20 Mother	3.423	2.351	2.603
Trio#21 ALS	3.332	2.350	2.578
Trio#21 Father	3.307	2.338	2.564
Trio#21 Mother	3.363	2.362	2.600
Trio#22 ALS	3.322	2.365	2.596
Trio#22 Father	3.312	2.376	2.603
Trio#22 Mother	3.292	2.372	2.596
Trio#23 ALS	3.252	2.320	2.536
Trio#23 Father	3.310	2.357	2.581
Trio#23 Mother	3.216	2.334	2.545
Trio#24 ALS	3.288	2.336	2.561
Trio#24 Father	3.301	2.359	2.581
Trio#24 Mother	3.272	2.343	2.562
Trio#25 ALS	3.287	2.344	2.560
Trio#25 Father	3.239	2.358	2.565
Trio#25 Mother	3.298	2.362	2.583
Trio#26 ALS	3.240	2.348	2.560
Trio#26 Father	3.262	2.324	2.542
Trio#26 Mother	3.279	2.339	2.558
Trio#27 ALS	3.256	2.348	2.561
Trio#27 Father	3.312	2.335	2.564
Trio#27 Mother	3.304	2.346	2.574

Trio#28 ALS	3.254	2.348	2.559
Trio#28 Father	3.275	2.320	2.536
Trio#28 Mother	3.293	2.339	2.558
Trio#29 ALS	3.240	2.346	2.559
Trio#29 Father	3.251	2.348	2.560
Trio#29 Mother	3.300	2.323	2.549
Trio#30 ALS	3.265	2.370	2.585
Trio#30 Father	3.323	2.364	2.589
Trio#30 Mother	3.266	2.346	2.568
Trio#31 ALS	3.266	2.346	2.567
Trio#31 Father	3.308	2.341	2.571
Trio#31 Mother	3.274	2.371	2.590
Trio#32 ALS	3.271	2.347	2.561
Trio#32 Father	3.261	2.338	2.551
Trio#32 Mother	3.265	2.339	2.554
Trio#33 ALS	3.316	2.347	2.575
Trio#33 Father	3.270	2.333	2.550
Trio#33 Mother	3.329	2.337	2.567
Trio#34 ALS	3.318	2.359	2.589
Trio#34 Father	3.384	2.353	2.600
Trio#34 Mother	3.333	2.348	2.582
Trio#35 ALS	3.267	2.328	2.548
Trio#35 Father	3.276	2.324	2.547
Trio#35 Mother	3.315	2.330	2.553
Trio#36 ALS	3.333	2.340	2.575
Trio#36 Father	3.376	2.334	2.581
Trio#36 Mother	3.326	2.346	2.579
Trio#37 ALS	3.313	2.330	2.559
Trio#37 Father	3.251	2.342	2.556
Trio#37 Mother	3.325	2.341	2.569
Trio#38 ALS	3.310	2.382	2.606
Trio#38 Father	3.338	2.374	2.601
Trio#38 Mother	3.317	2.393	2.617
Trio#39 ALS	3.304	2.360	2.585
Trio#39 Father	3.271	2.348	2.567
Trio#39 Mother	3.308	2.354	2.576
Trio#40 ALS	3.345	2.384	2.617
Trio#40 Father	3.283	2.380	2.601
Trio#40 Mother	3.348	2.365	2.599
Trio#41 ALS	3.305	2.363	2.583
Trio#41 Father	3.299	2.353	2.575
Trio#41 Mother	3.297	2.348	2.575
Trio#42 ALS	3.209	2.336	2.544

Trio#42 Father	3.302	2.353	2.578
Trio#42 Mother	3.248	2.336	2.552
Trio#43 ALS	3.314	2.349	2.573
Trio#43 Father	3.283	2.347	2.567
Trio#43 Mother	3.268	2.352	2.567
Trio#44 ALS	3.283	2.342	2.562
Trio#44 Father	3.298	2.360	2.579
Trio#44 Mother	3.312	2.331	2.560

Supplementary Table S3. Replacement (amino acid change) to silent (no amino acid change) ratio per individual.

Trio ID	Total Rep COUNT	Het Rep	Hom Alt Rep	Het Rep + (Hom Alt Rep x2)	Total Sil COUNT	Het Sil	Hom Alt Sil	Het Sil + (Hom Alt Sil x2)	Rep:Sil ratio	Total variants (Both COUNTS)
Trio#01 ALS	8372	5197	3175	11547	6066	3674	2392	8458	1.3652	14438
Trio#01 Father	8362	5112	3250	11612	6221	3792	2429	8650	1.3424	14583
Trio#01 Mother	8303	5088	3215	11518	6097	3742	2355	8452	1.3628	14400
Trio#02 ALS	8386	5024	3362	11748	6121	3758	2363	8484	1.3847	14507
Trio#02 Father	8320	5012	3308	11628	6084	3709	2375	8459	1.3746	14404
Trio#02 Mother	8255	4884	3371	11626	6117	3740	2377	8494	1.3687	14372
Trio#03 ALS	8481	5216	3265	11746	6136	3830	2306	8442	1.3914	14617
Trio#03 Father	8251	5061	3190	11441	6028	3686	2342	8370	1.3669	14279
Trio#03 Mother	8283	5056	3227	11510	6185	3795	2390	8575	1.3423	14468
Trio#04 ALS	8253	4978	3275	11528	6153	3728	2425	8578	1.3439	14406
Trio#04 Father	8306	5022	3284	11590	6185	3745	2440	8625	1.3438	14491
Trio#04 Mother	8071	4760	3311	11382	6280	3927	2353	8633	1.3184	14351
Trio#05 ALS	8295	5047	3248	11543	6139	3754	2385	8524	1.3542	14434
Trio#05 Father	8308	5059	3249	11557	6136	3715	2421	8557	1.3506	14444
Trio#05 Mother	8666	5562	3104	11770	6221	3840	2381	8602	1.3683	14887
Trio#06 ALS	8624	5389	3235	11859	6028	3695	2333	8361	1.4184	14652
Trio#06 Father	8077	4822	3255	11332	6071	3756	2315	8386	1.3513	14148
Trio#06 Mother	8393	5233	3160	11553	6130	3769	2361	8491	1.3606	14523
Trio#07 ALS	8382	5058	3324	11706	6145	3683	2462	8607	1.3601	14527
Trio#07 Father	8370	5170	3200	11570	6084	3707	2377	8461	1.3675	14454
Trio#07 Mother	8547	5322	3225	11772	6159	3722	2437	8596	1.3695	14706
Trio#08 ALS	8361	5084	3277	11638	6141	3795	2346	8487	1.3713	14502
Trio#08 Father	8515	5297	3218	11733	6114	3672	2442	8556	1.3713	14629
Trio#08 Mother	8351	5107	3244	11595	6190	3809	2381	8571	1.3528	14541
Trio#09 ALS	8580	5404	3176	11756	6110	3665	2445	8555	1.3742	14690
Trio#09 Father	8344	5029	3315	11659	6114	3682	2432	8546	1.3643	14458
Trio#09 Mother	8301	5048	3253	11554	6146	3722	2424	8570	1.3482	14447
Trio#10 ALS	8439	5253	3186	11625	6052	3697	2355	8407	1.3828	14491
Trio#10 Father	8429	5118	3311	11740	6211	3768	2443	8654	1.3566	14640

Trio#10 Mother	8500	5259	3241	11741	6172	3843	2329	8501	1.3811	14672
Trio#11 ALS	8452	5202	3250	11702	6251	3926	2325	8576	1.3645	14703
Trio#11 Father	8311	5134	3177	11488	6115	3737	2378	8493	1.3526	14426
Trio#11 Mother	8377	5130	3247	11624	6244	3839	2405	8649	1.3440	14621
Trio#12 ALS	8160	4803	3357	11517	6121	3679	2442	8563	1.3450	14281
Trio#12 Father	8364	5054	3310	11674	6243	3822	2421	8664	1.3474	14607
Trio#12 Mother	8475	5293	3182	11657	6187	3850	2337	8524	1.3676	14662
Trio#13 ALS	8456	5266	3190	11646	6026	3574	2452	8478	1.3737	14482
Trio#13 Father	8379	5028	3351	11730	6123	3705	2418	8541	1.3734	14502
Trio#13 Mother	8388	5167	3221	11609	6100	3678	2422	8522	1.3622	14488
Trio#14 ALS	8384	5170	3214	11598	6076	3642	2434	8510	1.3629	14460
Trio#14 Father	8344	4879	3465	11809	6127	3784	2343	8470	1.3942	14471
Trio#14 Mother	8354	4984	3370	11724	6088	3701	2387	8475	1.3834	14442
Trio#15 ALS	8200	4871	3329	11529	6170	3735	2435	8605	1.3398	14370
Trio#15 Father	8525	5284	3241	11766	6150	3624	2526	8676	1.3562	14675
Trio#15 Mother	8178	4906	3272	11450	6185	3803	2382	8567	1.3365	14363
Trio#16 ALS	8354	5071	3283	11637	6189	3798	2391	8580	1.3563	14543
Trio#16 Father	8391	5082	3309	11700	6215	3871	2344	8559	1.3670	14606
Trio#16 Mother	8395	5075	3320	11715	6197	3813	2384	8581	1.3652	14592
Trio#17 ALS	8411	5106	3305	11716	6137	3749	2388	8525	1.3743	14548
Trio#17 Father	8164	4950	3214	11378	6186	3729	2457	8643	1.3164	14350
Trio#17 Mother	8331	5066	3265	11596	6103	3730	2373	8476	1.3681	14434
Trio#18 ALS	8338	5092	3246	11584	6244	3801	2443	8687	1.3335	14582
Trio#18 Father	8253	5014	3239	11492	6173	3672	2501	8674	1.3249	14426
Trio#18 Mother	8267	4979	3288	11555	6115	3745	2370	8485	1.3618	14382
Trio#19 ALS	8366	5136	3230	11596	6116	3735	2381	8497	1.3647	14482
Trio#19 Father	8530	5322	3208	11738	6143	3731	2412	8555	1.3721	14673
Trio#19 Mother	8443	5233	3210	11653	6218	3848	2370	8588	1.3569	14661
Trio#20 ALS	8336	5156	3180	11516	6018	3454	2564	8582	1.3419	14354
Trio#20 Father	7839	4718	3121	10960	6083	3683	2400	8483	1.2920	13922
Trio#20 Mother	8359	5185	3174	11533	6152	3686	2466	8618	1.3382	14511
Trio#21 ALS	8350	5003	3347	11697	6092	3697	2395	8487	1.3782	14442
Trio#21 Father	8308	4983	3325	11633	6037	3652	2385	8422	1.3813	14345
Trio#21 Mother	8410	5156	3254	11664	6081	3792	2289	8370	1.3935	14491
Trio#22 ALS	8155	4842	3313	11468	6017	3568	2449	8466	1.3546	14172
Trio#22 Father	8348	5152	3196	11544	6102	3749	2353	8455	1.3653	14450

Trio#22 Mother	8310	5009	3301	11611	6037	3605	2432	8469	1.3710	14347
Trio#23 ALS	8217	4936	3281	11498	6232	3821	2411	8643	1.3303	14449
Trio#23 Father	8123	4867	3256	11379	6224	3752	2472	8696	1.3085	14347
Trio#23 Mother	8415	5229	3186	11601	6172	3806	2366	8538	1.3587	14587
Trio#24 ALS	8281	5083	3198	11479	6335	4045	2290	8625	1.3309	14616
Trio#24 Father	7969	4567	3402	11371	6049	3502	2547	8596	1.3228	14018
Trio#24 Mother	8311	5017	3294	11605	6182	3818	2364	8546	1.3579	14493
Trio#25 ALS	8349	5100	3249	11598	6100	3777	2323	8423	1.3769	14449
Trio#25 Father	8420	5140	3280	11700	6091	3751	2340	8431	1.3877	14511
Trio#25 Mother	8461	5126	3335	11796	6223	3888	2335	8558	1.3784	14684
Trio#26 ALS	8350	4999	3351	11701	6037	3656	2381	8418	1.3900	14387
Trio#26 Father	8440	5190	3250	11690	6040	3688	2352	8392	1.3930	14480
Trio#26 Mother	8316	5099	3217	11533	6092	3640	2452	8544	1.3498	14408
Trio#27 ALS	8144	4859	3285	11429	6194	3825	2369	8563	1.3347	14338
Trio#27 Father	8393	5163	3230	11623	6102	3705	2397	8499	1.3676	14495
Trio#27 Mother	8447	5154	3293	11740	6148	3827	2321	8469	1.3862	14595
Trio#28 ALS	8443	5100	3343	11786	5983	3615	2368	8351	1.4113	14426
Trio#28 Father	8388	5153	3235	11623	5999	3706	2293	8292	1.4017	14387
Trio#28 Mother	8087	4729	3358	11445	6145	3770	2375	8520	1.3433	14232
Trio#29 ALS	8810	5882	2928	11738	6207	3800	2407	8614	1.3627	15017
Trio#29 Father	8468	5145	3323	11791	6124	3700	2424	8548	1.3794	14592
Trio#29 Mother	8379	5062	3317	11696	6248	3875	2373	8621	1.3567	14627
Trio#30 ALS	8500	5363	3137	11637	6138	3727	2411	8549	1.3612	14638
Trio#30 Father	8216	4967	3249	11465	6068	3673	2395	8463	1.3547	14284
Trio#30 Mother	8307	5070	3237	11544	6055	3671	2384	8439	1.3679	14362
Trio#31 ALS	8237	4966	3271	11508	6132	3796	2336	8468	1.3590	14369
Trio#31 Father	8173	4956	3217	11390	6129	3662	2467	8596	1.3250	14302
Trio#31 Mother	8524	5111	3413	11937	6120	3694	2426	8546	1.3968	14644
Trio#32 ALS	8375	5109	3266	11641	6143	3787	2356	8499	1.3697	14518
Trio#32 Father	8504	5240	3264	11768	6174	3767	2407	8581	1.3714	14678
Trio#32 Mother	8327	5085	3242	11569	6203	3792	2411	8614	1.3430	14530
Trio#33 ALS	8270	5079	3191	11461	6117	3827	2290	8407	1.3633	14387
Trio#33 Father	8416	5145	3271	11687	6150	3765	2385	8535	1.3693	14566
Trio#33 Mother	8275	5006	3269	11544	6040	3662	2378	8418	1.3713	14315
Trio#34 ALS	8610	5393	3217	11827	6053	3685	2368	8421	1.4045	14663
Trio#34 Father	8378	5124	3254	11632	6155	3736	2419	8574	1.3567	14533

Trio#34 Mother	8465	5195	3270	11735	6101	3688	2413	8514	1.3783	14566
Trio#35 ALS	8407	5145	3262	11669	6112	3760	2352	8464	1.3787	14519
Trio#35 Father	8330	5090	3240	11570	6121	3716	2405	8526	1.3570	14451
Trio#35 Mother	8284	5129	3155	11439	6159	3759	2400	8559	1.3365	14443
Trio#36 ALS	8424	5064	3360	11784	6215	3936	2279	8494	1.3873	14639
Trio#36 Father	8306	4871	3435	11741	6178	3879	2299	8477	1.3850	14484
Trio#36 Mother	7802	4612	3190	10992	6090	3744	2346	8436	1.3030	13892
Trio#37 ALS	8563	5337	3226	11789	6288	3857	2431	8719	1.3521	14851
Trio#37 Father	8251	5009	3242	11493	6104	3754	2350	8454	1.3595	14355
Trio#37 Mother	8242	4984	3258	11500	6309	3852	2457	8766	1.3119	14551
Trio#38 ALS	8405	5194	3211	11616	6117	3695	2422	8539	1.3603	14522
Trio#38 Father	8413	5214	3199	11612	6067	3637	2430	8497	1.3666	14480
Trio#38 Mother	8280	5026	3254	11534	6194	3835	2359	8553	1.3485	14474
Trio#39 ALS	8385	5182	3203	11588	6202	3770	2432	8634	1.3421	14587
Trio#39 Father	8295	4996	3299	11594	6169	3740	2429	8598	1.3485	14464
Trio#39 Mother	8171	5043	3128	11299	6280	3874	2406	8686	1.3008	14451
Trio#40 ALS	8173	4907	3266	11439	6033	3667	2366	8399	1.3619	14206
Trio#40 Father	8267	5044	3223	11490	6093	3730	2363	8456	1.3588	14360
Trio#40 Mother	8274	5040	3234	11508	6065	3748	2317	8382	1.3729	14339
Trio#41 ALS	8382	5130	3252	11634	6232	3912	2320	8552	1.3604	14614
Trio#41 Father	8418	5123	3295	11713	6111	3765	2346	8457	1.3850	14529
Trio#41 Mother	8317	5021	3296	11613	6216	3909	2307	8523	1.3625	14533
Trio#42 ALS	8249	5094	3155	11404	6222	3843	2379	8601	1.3259	14471
Trio#42 Father	8291	5010	3281	11572	6186	3806	2380	8566	1.3509	14477
Trio#42 Mother	8412	5245	3167	11579	6276	3937	2339	8615	1.3441	14688
Trio#43 ALS	8223	4913	3310	11533	6055	3733	2322	8377	1.3767	14278
Trio#43 Father	8401	5191	3210	11611	6078	3755	2323	8401	1.3821	14479
Trio#43 Mother	8456	5184	3272	11728	6079	3693	2386	8465	1.3855	14535
Trio#44 ALS	8462	5156	3306	11768	6177	3806	2371	8548	1.3767	14639
Trio#44 Father	8067	4783	3284	11351	6193	3814	2379	8572	1.3242	14260
Trio#44 Mother	8321	4926	3395	11716	6226	3912	2314	8540	1.3719	14547

Supplementary Table S4: All coding and non-coding rare de novo variants detected in ALS trio patients

Gene	Trio ID	Chr	Position	Impact	MAF ESP	MAF 1KG	rsID
AGER	Trio#35 ALS	6	32148724	downstream	None	0.02	rs41270464
AKD1	Trio#08 ALS	6	109894726	non_syn_coding	None	None	None
ANAPC7	Trio#21 ALS	12	110819574	non_syn_coding	None	None	None
ARID4B	Trio#13 ALS	1	235385028	intron	None	None	None
C6orf201	Trio#35 ALS	6	4130313	downstream	0.212659	0.17	rs13210282
CACNA2D2	Trio#16 ALS	3	50511592	intron	None	None	None
CDYL	Trio#35 ALS	6	4954710	downstream	None	0.61	rs55682129
CHRM1	Trio#41 ALS	11	62678572	start_loss	None	None	None
CNST	Trio#32 ALS	1	246797141	intron	None	None	None
COL11A2	Trio#35 ALS	6	33143948	downstream	None	0.54	rs2254287
DALRD3	Trio#34 ALS	3	49049038	downstream	None	None	None
ERG	Trio#41 ALS	21	39764329	intron	0.000385	0.0009	rs18975523
FBXO33	Trio#21 ALS	14	39867173	UTR_3_prime	None	None	None
FOXN3	Trio#30 ALS	14	89656737	stop_gain	None	None	None
FZD5	Trio#43 ALS	2	208627504	UTR_3_prime	None	None	None
GCFC1	Trio#02 ALS	21	34132207	synonymous_coding	None	None	None
GTF2H4	Trio#35 ALS	6	30880156	non_syn_coding	0.010308	0.01	rs3218820
HNRNPK	Trio#41 ALS	9	86589394	intron	None	None	None
IRF4	Trio#35 ALS	6	405085	synonymous_coding	0.000077	None	rs138866854
ITPR2	Trio#42 ALS	12	26808680	non_syn_coding	None	None	None
JARID2	Trio#35 ALS	6	15513436	intron	None	None	None
KLF13	Trio#10 ALS	15	31666253	UTR_3_prime	None	None	None
LIMCH1	Trio#14 ALS	4	41648333	intron	None	None	None
LIMD1	Trio#02 ALS	3	45637047	non_syn_coding	None	None	None
LRBA	Trio#23 ALS	4	151417047	intron	None	None	None

MCIN	Trio#26 ALS	5	54516341	synonymous_coding	None	None	None
METTL22	Trio#30 ALS	16	8738455	non_syn_coding	None	None	None
MLL3	Trio#02 ALS	7	151849993	non_syn_coding	None	None	None
MORN5	Trio#24 ALS	9	124936621	intron	None	None	None
MUC16	Trio#23 ALS	19	9083943	synonymous_coding	None	None	None
MYLK4	Trio#35 ALS	6	2717355	intron	None	0.18	rs4959712
NLRC5	Trio#43 ALS	16	57073761	non_syn_coding, splice	None	None	None
None	Trio#15 ALS	14	82128930	None	None	None	None
None	Trio#23 ALS	2	189092702	None	None	None	None
PLA2G4C	Trio#24 ALS	19	48607867	non_syn_coding	None	None	rs13895674
PSMB7	Trio#08 ALS	9	127119118	non_syn_coding	None	None	None
RBFOX1	Trio#35 ALS	16	7761443	UTR_3_prime	None	None	None
RINL	Trio#10 ALS	19	39359972	non_syn_coding	None	None	None
RPL4	Trio#40 ALS	15	66794068	downstream	None	None	None
SEC14L1	Trio#17 ALS	17	75209286	intron	None	None	None
SND1	Trio#24 ALS	7	127341354	non_syn_coding	None	None	rs34667910
SRP14	Trio#36 ALS	15	40325033	downstream	None	None	None
STK36	Trio#14 ALS	2	219538460	non_syn_coding	None	None	None
SV2A	Trio#20 ALS	1	149885128	non_syn_coding	None	None	None
TCIRG1	Trio#23 ALS	11	67821491	downstream	None	None	None
TEKT3	Trio#03 ALS	17	15217439	synonymous_coding	0.000077	0.0005	rs200743438
TMEM180	Trio#16 ALS	10	104241912	downstream	None	None	None
TMEM182	Trio#05 ALS	2	103378664	intron	None	None	None
TRPA1	Trio#05 ALS	8	72950332	intron	None	None	None
TRRAP	Trio#35 ALS	7	98553842	non_syn_coding	None	None	None
VPS39	Trio#02 ALS	15	42479962	synonymous_coding	None	None	None
XKR4	Trio#43 ALS	8	56364311	intron	None	None	None
YEATS2	Trio#26 ALS	3	183529237	UTR_3_prime	None	None	None
ZBTB46	Trio#30 ALS	20	62384144	synonymous_coding	None	None	None

Supplementary Table S5: Coding variants in ALSoD genes in ALS trio patients

Gene	Chr	Position	Impact	MAF ESP	MAF 1KG	MAF unaffected carrier	MAF affected carrier
ALS2	2	202625615	non_syn_coding	0.1386	0.9000	0.9896	0.9792
ALS2	2	202625992	non_syn_coding	None	None	0.0104	0.0000
ALS2	2	202626437	non_syn_coding	0.0335	0.0200	0.0729	0.0417
ATXN2	12	111902514	non_syn_coding	0.0012	0.0009	0.0104	0.0000
ATXN2	12	111908545	non_syn_coding	0.0032	0.0023	0.0208	0.0208
ATXN2	12	111993712	non_syn_coding	0.0431	0.2000	0.0104	0.0208
BCL11B	14	99642265	non_syn_coding	None	None	0.0104	0.0208
BCL6	3	187446211	non_syn_coding	0.2377	0.1800	0.3854	0.4583
BCL6	3	187447565	non_syn_coding	0.0001	None	0.0104	0.0000
BCL6	3	187447701	non_syn_coding	0.0241	0.0200	0.0625	0.0417
C9orf72	9	27556780	non_syn_coding	0.1962	0.2100	0.2604	0.2708
C9orf72	9	27561628	non_syn_coding	0.0950	0.0500	0.1979	0.1875
C9orf72	9	27567145	start_gain	0.2080	0.1800	0.3958	0.3958
CDH13	16	82892037	non_syn_coding	0.0149	0.0300	0.0208	0.0208
CDH13	16	83704419	non_syn_coding	0.0053	0.0037	0.0208	0.0417
CDH13	16	83813644	non_syn_coding	0.0005	None	0.0104	0.0208
CDH13	16	83816871	non_syn_coding	0.0041	0.0041	0.0208	0.0208
CDH22	20	44806808	non_syn_coding	0.0017	0.0014	0.0208	0.0417
CDH22	20	44815281	non_syn_coding	None	None	0.0104	0.0000
CDH22	20	44815292	non_syn_coding	0.0025	0.0018	0.0104	0.0208
CHMP2B	3	87276571	start_gain	None	0.0046	0.0104	0.0208
CHMP2B	3	87276699	start_gain	0.0935	0.1200	0.1354	0.1667
CNTN6	3	1262474	start_gain	0.3815	0.4300	0.6250	0.5625
CNTN6	3	1337325	stop_gain	None	None	0.0104	0.0000

CNTN6	3	1337342	non_syn_coding	None	None	0.0104	0.0208
CNTN6	3	1363480	non_syn_coding	0.0070	0.0027	0.0208	0.0208
CNTN6	3	1363500	non_syn_coding	0.0012	0.0005	0.0104	0.0000
CNTN6	3	1394056	non_syn_coding	None	None	0.0104	0.0208
CNTN6	3	1414110	non_syn_coding	None	None	0.0104	0.0208
CNTN6	3	1418754	non_syn_coding	None	None	0.0104	0.0208
CRIM1	2	36583461	non_syn_coding	0.0046	0.0700	0.0417	0.0417
CRIM1	2	36706700	non_syn_coding	0.0022	None	0.0104	0.0000
CRIM1	2	36737128	non_syn_coding	0.0098	0.0100	0.0104	0.0417
CRIM1	2	36774132	non_syn_coding	0.0001	None	0.0208	0.0208
CRYM	16	21289465	non_syn_coding	0.0002	None	0.0104	0.0000
DAO	12	109278969	non_syn_coding	0.0002	None	0.0104	0.0000
DAO	12	109281299	non_syn_coding	0.0003	None	0.0104	0.0208
DCTN1	2	74596527	non_syn_coding	0.0138	0.0100	0.0313	0.0625
DCTN1	2	74598723	non_syn_coding	0.0055	0.0027	0.0313	0.0417
DIAPH3	13	60490311	non_syn_coding	None	None	0.0104	0.0208
DIAPH3	13	60545105	non_syn_coding	0.0046	0.0018	0.0104	0.0000
DIAPH3	13	60545182	non_syn_coding	0.0307	0.0200	0.0938	0.1667
DIAPH3	13	60565291	splice_donor	None	None	0.0104	0.0000
DIAPH3	13	60566644	non_syn_coding	0.0668	0.0600	0.1042	0.0833
DIAPH3	13	60737860	non_syn_coding	0.0001	None	0.0104	0.0000
EWSR1	22	29693915	non_syn_coding	0.0097	0.0046	0.0104	0.0208
FIG4	6	110012507	start_gain	None	None	0.0208	0.0417
FIG4	6	110064928	non_syn_coding	0.0309	0.1000	0.0625	0.0625
FIG4	6	110107517	non_syn_coding	0.3267	0.3700	0.2292	0.2292
FUS	16	31191485	start_gain	None	None	0.0104	0.0000
GRB14	2	165381542	non_syn_coding	0.0162	0.0100	0.0208	0.0000
GRB14	2	165404195	non_syn_coding	0.0001	None	0.0104	0.0208
GRB14	2	165476253	non_syn_coding	0.4762	0.5000	0.8750	0.8542
LUM	12	91502161	non_syn_coding	0.0033	0.0018	0.0104	0.0208

LUM	12	91502176	non_syn_coding	None	None	0.0104	0.0000
NEFH	22	29879534	non_syn_coding	0.0035	0.0014	0.0104	0.0208
NEFH	22	29885016	non_syn_coding	0.0702	0.0400	0.1875	0.1875
NEFH	22	29885473	non_syn_coding	0.2010	0.1700	0.3854	0.2500
NEFH	22	29886043	non_syn_coding	0.1446	0.1200	0.3021	0.4375
NETO1	18	70417396	non_syn_coding	0.0105	0.9900	1.0000	1.0000
NETO1	18	70417409	non_syn_coding	0.0103	0.0100	0.0208	0.0208
NETO1	18	70417634	non_syn_coding	0.0023	0.0005	0.0104	0.0000
OMA1	1	58946647	non_syn_coding	0.0014	None	0.0313	0.0833
OMA1	1	58996320	non_syn_coding	0.0035	0.0023	0.0104	0.0208
OMA1	1	58999651	non_syn_coding	0.1309	0.1000	0.1563	0.0833
OMA1	1	59002282	non_syn_coding	0.0113	0.0400	0.0208	0.0208
OMA1	1	59004617	non_syn_coding	0.0089	0.0400	0.0208	0.0208
OMA1	1	59004979	start_gain	0.0249	0.9800	1.0000	1.0000
OPTN	10	13152400	non_syn_coding	0.0611	0.0700	0.0521	0.0625
OPTN	10	13166076	non_syn_coding	0.0094	0.9900	1.0000	1.0000
OPTN	10	13174076	non_syn_coding	None	None	0.0104	0.0208
RAMP3	7	45217015	non_syn_coding	0.0484	0.0600	0.1042	0.0833
RAMP3	7	45222817	non_syn_coding	0.0004	None	0.0104	0.0208
RAMP3	7	45222912	non_syn_coding	0.0003	None	0.0104	0.0000
SETX	9	135139826	non_syn_coding	0.0730	0.1600	0.0625	0.1042
SETX	9	135139901	non_syn_coding	0.4442	0.5100	0.4896	0.6458
SETX	9	135140020	non_syn_coding	0.0054	0.0027	0.0208	0.0417
SETX	9	135173685	non_syn_coding	0.3068	0.4100	0.2708	0.3542
SETX	9	135202829	non_syn_coding	0.3062	0.5900	0.9792	0.9583
SETX	9	135203409	non_syn_coding	0.2071	0.6600	0.9792	0.9583
SETX	9	135203530	non_syn_coding	0.0384	0.1000	0.0313	0.0417
SETX	9	135204010	non_syn_coding	0.0117	0.0100	0.0313	0.0208
SETX	9	135204688	non_syn_coding	None	None	0.0104	0.0208
SETX	9	135205006	non_syn_coding	0.1138	0.2100	0.0729	0.1042

SETX	9	135218103	non_syn_coding	0.0040	0.0005	0.0104	0.0000
SETX	9	135224757	non_syn_coding	0.0075	0.0100	0.0313	0.0000
SOD1	21	33031974	start_gain	None	0.0300	0.0104	0.0208
SOD1	21	33039672	non_syn_coding	None	None	0.0104	0.0208
SPG11	15	44859744	non_syn_coding	0.0012	None	0.0104	0.0000
SPG11	15	44912566	non_syn_coding	0.0038	0.0032	0.0104	0.0208
SPG11	15	44918690	non_syn_coding	0.0097	0.0100	0.0521	0.0208
SPG11	15	44925740	non_syn_coding	0.0133	0.0100	0.0417	0.0417
SPG11	15	44943710	non_syn_coding	None	None	0.0104	0.0208
SPG11	15	44943757	non_syn_coding	0.4793	0.4900	0.6563	0.6458
SPG11	15	44944037	non_syn_coding	0.0146	0.0100	0.0417	0.0208
SPG11	15	44949354	non_syn_coding	0.0102	0.0037	0.0104	0.0208
SQSTM1	5	179252184	non_syn_coding	0.0026	0.0032	0.0104	0.0208
SQSTM1	5	179260099	non_syn_coding	0.0187	0.0100	0.0521	0.0417
SQSTM1	5	179263445	non_syn_coding	0.0015	0.0046	0.0104	0.0000
SYT9	11	7324584	non_syn_coding	0.0270	0.0200	0.1042	0.0833
SYT9	11	7335424	non_syn_coding	None	0.3900	0.8125	0.8542
SYT9	11	7437285	non_syn_coding	0.0080	0.0027	0.0417	0.0625
TAF15	17	34147222	non_syn_coding	0.0010	0.0009	0.0104	0.0000
TAF15	17	34171358	non_syn_coding	0.0005	None	0.0104	0.0000

Supplementary Table S6: Sequencing metrics

	Mean (SD)
Total reads (billions)	9.12 (5.02)
% reads aligned	98.5
% duplicate reads	4.19
Average coverage	52.5X (12)
% on target bases	59.8
% target at 5X	96.96 (1.18)
% target at 10X	94.92 (2.18)
% target at 20X	86.98 (5.08)
Ts/Tv all	2.601
Ts/Tv coding	3.261
Ts/Tv non-coding	2.377
Average % of coding bases covered	≥20
Average no. variants per individual	64,874 (SD 1,621)
Range of variants	60,271 – 68,241
% variants in dbSNP137 or NHLBI ESP	83

Supplementary Methods

Exome sequencing

Genomic DNA from peripheral blood nucleated cells was extracted using QIAmp 96 DNA Blood Kits (Qiagen, Hilden, Germany). The integrity and yield of native genomic DNA was verified by a PicoGreen assay for quantitation and run on a 0.8% agarose gel for a qualitative QC. Illumina paired end small fragment libraries were constructed according to the manufacturer's recommendations with the following exceptions: (1) 500-1000 ng of native genomic DNA was fragmented using the Covaris E220 DNA Sonicator to a size range between 100-400 bp, (2) Illumina adapter-ligated library fragments were amplified in four 50 µL PCR reactions for eighteen cycles, (3) Solid Phase Reversible Immobilization (SPRI) bead clean-up was used for enzymatic purification throughout the library process, as well as final library size selection targeting 300-500 bp fragments.

Libraries were pooled pre-capture and hybridized to NimbleGen SeqCap EZ Human Exome Library kits (Roche NimbleGen, Madison, WI) according to manufacturer's protocol. Thirty samples were pooled to 4 libraries per pool and were processed on the v2.0 kits; 102 samples were pooled (5 libraries per pool) and processed on the v3.0 kits. The concentration of each captured library was accurately determined through KAPA qPCR according to the manufacturer's protocol to produce cluster counts appropriate for the Illumina HiSeq 2000 platform. The libraries were run on a HiSeq2000 V3 2x101 bp sequencing run according to manufacturer recommendations. Sequence data were aligned to the GRCh37-lite reference sequence using bwa version 0.5.9[1]. The targeted bases were defined by the v2.0 library kits, and 80% of targeted bases at 20X were required for the sample to pass quality control (QC). If Illumina OmniExpress genotype array data were available the sequencing data were also compared to the SNP calls. A 90% concordance rate was required for the sample to pass QC.

Variant calling and annotation pipelines

Variants were called using Samtools version r963 and VarScan 2.2.9[2]. Variants were filtered using read count information from the bam files which required a minimum base quality of 15, a maximum difference of mapping quality between variant and reference reads equal to 30, and a maximum difference of average supporting read length of 25, in addition to filters regarding homopolymer stretches, strandedness and minimum variant allele frequency. We restricted variant calling in all samples to a region of interest set that contains 34Mbp of consensus coding sequence, defined from the overlap between various versions of NimbleGen and Agilent exome capture reagents originally used in the TCGA project[3]. A 500 bp wingspan was added to each target region of interest on each side that adds an additional ~200 kbp of non-coding space. The dataset contained the unique union of the filtered calls from Samtools and VarScan, and genotype calls were combined from all individuals into a multisample vcf file. The site was removed if the false positive filter flagged 50% or more of the genotypes at a given position.

Familial relationships were verified using BEAGLE's fastIBD tool[4] to calculate the identity by descent between the child and expected parents. If a parent-child pair shared fewer than 40% of variants the entire trio was excluded.

A pedigree aware calling method, Polymutt[5], that employs a likelihood-based framework, was utilised to call *de novo* variants in the trios. This method provides increased sensitivity and specificity when calling *de novo* point mutations by leveraging the parental genotype information when making calls.

The final variant set was annotated with dbSNP137 and Variant Effect Predictor (VEP) version 2.2[6] with the following parameters: *--condel b --polyphen b --sift b --hgnc --canonical*. For each gene, the canonical VEP annotation was used whenever possible. In the

event of multiple overlapping genes that yielded different annotations, the most damaging annotation was used. Trio phasing was performed using BEAGLE v4 with default settings[7]. Variants that intersected with sequence annotated in RepeatMasker and segmental duplications were excluded. All *de novo* variants were evaluated regardless of functional impact.

Two software programs were used to further annotate and aid in prioritizing variants, (A) Ingenuity Variant Analysis (IVA; www.ingenuity.com/variants) and (B) GEMINI v.0.6.3[8]. (A) IVA employs a series of filters to assess functional consequences and impact of variants. Autosomal recessive and compound heterozygous variants were filtered with: (1) *high confidence*, read depth ≥ 20 and variants outside the top 1% of most exonically variable genes in healthy individuals, (2) *rare variants*, less than 1% frequency in Europeans from 1000 Genomes Project, NHLBI Exome Sequencing Project and Complete Genomics data, (3) *predicted deleterious*, predicted pathogenic or likely pathogenic, predicted gain of function, predicted loss of function (missense not tolerated by SIFT or PolyPhen2; frameshift, stop codon or essential splice site), (4) *inheritance*, homozygous, compound heterozygous, hemizygous, or haploinsufficient in cases but not controls, (5) *affect motor neurons*, diseases associated with motor neuron degradation, apoptosis, atrophy, demyelination, deficiency and damage. (B) Autosomal recessive and compound heterozygous variants were prioritized as candidates using GEMINI if they were: (1) *coding*, (2) *rare*, with a < 1% minor allele frequency in the 1000 Genomes and NHLBI Exome Sequencing projects, (3) *deleterious or damaging*, using SIFT, PolyPhen and Condel, and (4) *highly conserved*, using GERP. The unique union of the IVA and GEMINI variants was used as the list of candidate variants for manual review.

Candidate variants were manually reviewed using the Integrative Genome Viewer (IGV)[9] and eliminated from further analysis if reads supported mapping errors due to paralogous sequences, or in the case of *de novo* variants, if there was evidence for the variant in one of the parents.

Validation of variants

Candidate variants that passed manual review were independently validated with 3730 Sanger sequencing. Primers were designed using an in-house software program and tailed for 3730 sequencing. The amplification reaction consisted of a 5 ng DNA input, primer pairs, and the Amplitaq enzyme and amplified for 3 h in a thermocycler. After amplification, the product was cleaned up using a 1.5:1.0 Ampure bead-to-sample ratio. A Lonza flash gel was run to confirm product. Two sequencing reactions were completed using a 2 μL DNA input, Big Dye, and either the forward or reverse universal primer. Once complete, the sequencing reaction was precipitated using sodium acetate followed by a 70% ethanol wash. The DNA was dried down and then resuspended in EDTA and loaded on the 3730 sequencing platform.

References

1. Li, H., Durbin, R. Fast and accurate short read alignment with Burrows-Wheeler transform. *Bioinformatics* **25**, 1754-1760 (2009).
2. Koboldt, D.C.*, et al.* VarScan: variant detection in massively parallel sequencing of individual and pooled samples. *Bioinformatics* **25**, 2283-2285 (2009).
3. Cancer Genome Atlas Research, N. Integrated genomic analyses of ovarian carcinoma. *Nature* **474**, 609-615 (2011).
4. Browning, B.L., Browning, S.R. A fast, powerful method for detecting identity by descent. *Am. J. Hum. Genet.* **88**, 173-182 (2011).
5. Li, B.*, et al.* A likelihood-based framework for variant calling and de novo mutation detection in families. *PLoS Genet.* **8**, e1002944 (2012).

6. Mclaren, W., Pritchard, B., Rios, D., Chen, Y., Flicek, P., Cunningham, F. Deriving the consequences of genomic variants with the Ensembl API and SNP Effect Predictor. *Bioinformatics* **26**, 2069-2070 (2010).
7. Browning, B.L., Browning, S.R. Improving the accuracy and efficiency of identity-by-descent detection in population data. *Genetics* **194**, 459-471 (2013).
8. Paila, U., Chapman, B.A., Kirchner, R., Quinlan, A.R. GEMINI: integrative exploration of genetic variation and genome annotations. *PLoS Comput. Biol.* **9**, e1003153 (2013).
9. Robinson, J.T*., et al.* Integrative genomics viewer. *Nat. Biotechnol.* **29**, 24-26 (2011).
